# Design, synthesis, and biological evaluation of 2,4-dimorpholinopyrimidine-5-carbonitrile derivatives as orally bioavailable PI3K inhibitors

**DOI:** 10.3389/fphar.2024.1467028

**Published:** 2024-10-21

**Authors:** Daowei Huang, Jixia Yang, Qingwei Zhang, Xiaolei Zhou, Yanbo Wang, Zhenhua Shang, Jianqi Li, Baoyin Zhang

**Affiliations:** ^1^ School of Chemical and Pharmaceutical Engineering, Hebei University of Science and Technology, Shijiazhuang, China; ^2^ Hebei Research Center of Pharmaceutical and Chemical Engineering, Shijiazhuang, China; ^3^ State Key Laboratory Breeding Base-Hebei Key Laboratory of Molecular Chemistry for Drug, Shijiazhuang, China; ^4^ School of Pharmacy, Hebei University of Chinese Medicine, Shijiazhuang, China; ^5^ Novel Technology Center of Pharmaceutical Chemistry, Shanghai Institute of Pharmaceutical Industry Co., Ltd., China State Institute of Pharmaceutical Industry, Shanghai, China; ^6^ School of Food Science and Biology, Hebei University of Science and Technology, Shijiazhuang, China; ^7^ Shijiazhuang Vortech Biotech Co., Ltd., Shijiazhuang, China; ^8^ Department of Pharmacy, The Third Affiliated Hospital of Zhengzhou University, Zhengzhou, China

**Keywords:** PI3Ks, 2, 4-dimorpholinopyrimidine-5-carbonitrile, synthesis, anti-cancer, bioavailability

## Abstract

**Introduction:**

Phosphoinositide-3-kinase (PI3K) is overexpressed in many tumors and is, thus, an ideal target for cancer treatments. Accordingly, there is an urgent need for the development of PI3K inhibitors with high potency and low toxicity.

**Methods:**

In this study, we designed and synthesized a series of 2,4-dimorpholinopyrimidine-5-carbonitrile derivatives, which were evaluated for their PI3K inhibitory potency.

**Results and discussion:**

Compound 17p demonstrated comparable PI3Kα inhibitory activity (IC_50_: 31.8 ± 4.1 nM) to the positive control, BKM-120 (IC_50_: 44.6 ± 3.6 nM). In addition, 17p showed significant inhibitory activity against PI3Kδ (IC_50_: 15.4 ± 1.9 nM) and significant isoform selectivity against PI3Kβ, PI3Kγ, and mTOR. Furthermore, 17p exhibited good antiproliferative activities against cancer cell activity and good safety in the Ames and hERG tests while having outstanding liver microsomal stability *in vitro*, with half-lives of 38.5 min in rats and 127.9 min in humans. In addition, in an apoptosis assay, 17p could induce dose-dependent cytotoxicity in the ovarian cancer cell line A2780. In a pharmacokinetic study, 17p was stable (*T*_½_: 2.03 h) and showed high bioavailability (46.2%). Collectively, these results indicate that 17p could be a promising PI3K agent for cancer treatment.

## 1 Introduction

Phosphoinositide 3-kinases (PI3Ks) belong to a family of lipid kinases that play a key role in cell growth, survival, migration, and differentiation (Bai et al., 2023; Xie et al., 2024; Cantley, 2002; Donahue et al., 2012). Accumulating evidence indicates that their dysregulation is implicated in the pathophysiology of various diseases, such as cancer, diabetes, and cardiovascular diseases. PI3Ks can be divided into three classes (I–III) (Zhang et al., 2023; Zhang et al., 2024; Donahue et al., 2012; Katso et al., 2001), according to their substrate specificity, sequence homology, and regulatory subtype. Class I, the most extensively studied subtype, can be further subdivided into classes IA (α, β, and δ isoforms) and IB (γ isoform) based on structural similarity and coupling. *In vivo,* class I PI3Ks mainly catalyze the phosphorylation of phosphatidylinositol (3,4)-bisphosphate (PIP2) to phosphatidylinositol (3,4,5)-triphosphate (PIP3) at position 3 of the inositol ring (Arendt et al., 2023; Vanhaesebroeck et al., 2021; Manning and Cantley, 2007), triggering a series of downstream effectors mediating cellular functions as a second messenger, something strictly controlled by the tumor suppressor phosphatase and tensin homolog (PTEN) (Hennessy et al., 2005). Meanwhile, class II and III PI3Ks catalyze the phosphorylation of other related phosphatidylinositol lipid substrates. Overexpression and mutations in class I PI3Ks have been identified in tumors such as lymphoma, prostate cancer, and gastric cancer (De Santis et al., 2019). Thus, extensive research is needed to develop candidates targeting the PI3K pathway for cancer treatment.

**FIGURE 1:**
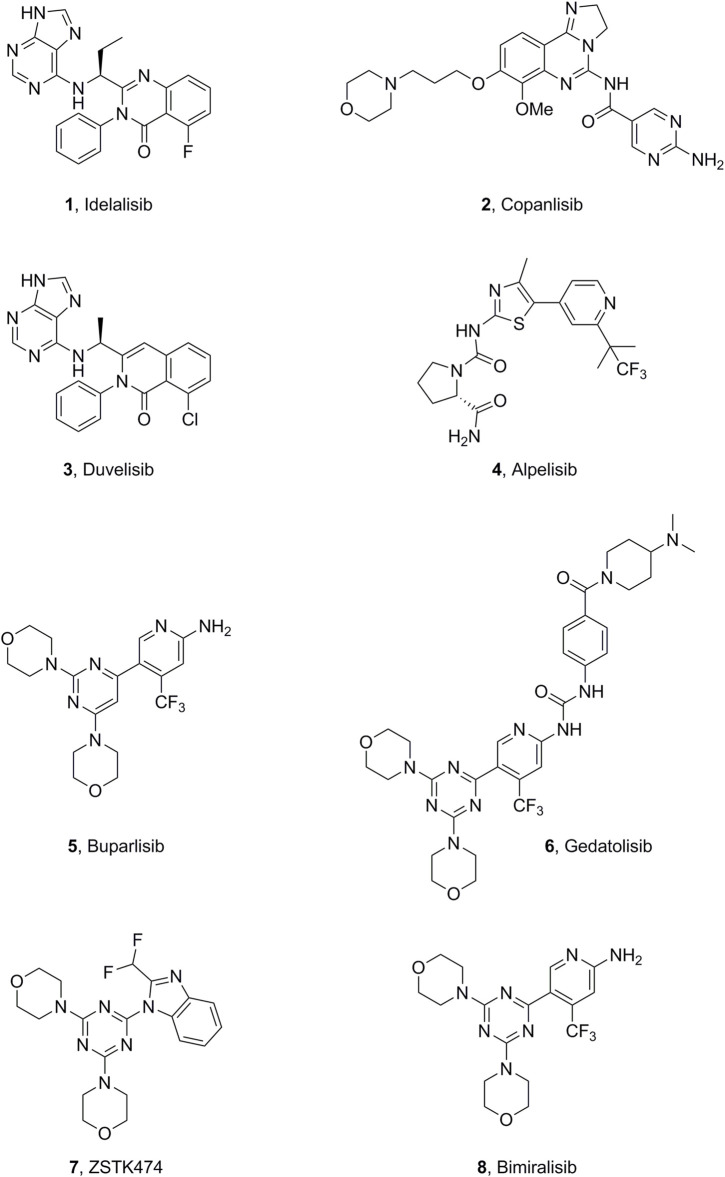
Representative small-molecule PI3K inhibitors.

PI3K inhibitors have been widely studied; four drugs (idelalisib, copanlisib, duvelisib, and alpelisib) have already been approved by the FDA (Figure 1). Idelalisib (CAL-101) (Mattsson et al., 2023; Markham, 2014), the first PI3Kδ selective inhibitor, was approved by the FDA in 2014, with indications for follicular B-cell non-Hodgkin lymphoma (FL) and relapsed small lymphocytic lymphoma. In 2017, copanlisib (Markham, 2017) was approved as a pan-PI3K inhibitor for adult patients with relapsing follicular lymphoma after ≥2 prior systemic therapies. The oral PI3Kδ/γ inhibitor duvelisib was approved for chronic lymphocytic leukemia, small lymphocytic lymphoma, and FL after other treatments (Till et al., 2023; Blair, 2018). The combination therapy of fulvestrant plus the PI3Kα-selective inhibitor alpelisib was approved in 2018 to treat advanced or metastatic breast cancer associated with hormone receptor-positive, human epidermal growth factor receptor 2-negative, and PI3KCA mutation (Copur and Jonglertham, 2019). Buparlisib (BKM-120) is under phase III clinical trials for the treatment of head and neck squamous cell carcinoma (HNSCC) (Martín et al., 2017). Moreover, there are many PI3K inhibitors and drug candidates under clinical and preclinical investigation (Garces and Stocks, 2018; Turner et al., 2020), such as bimiralisib, ZSTK474, and gedatolisib (Figure 1).

**FIGURE 2:**
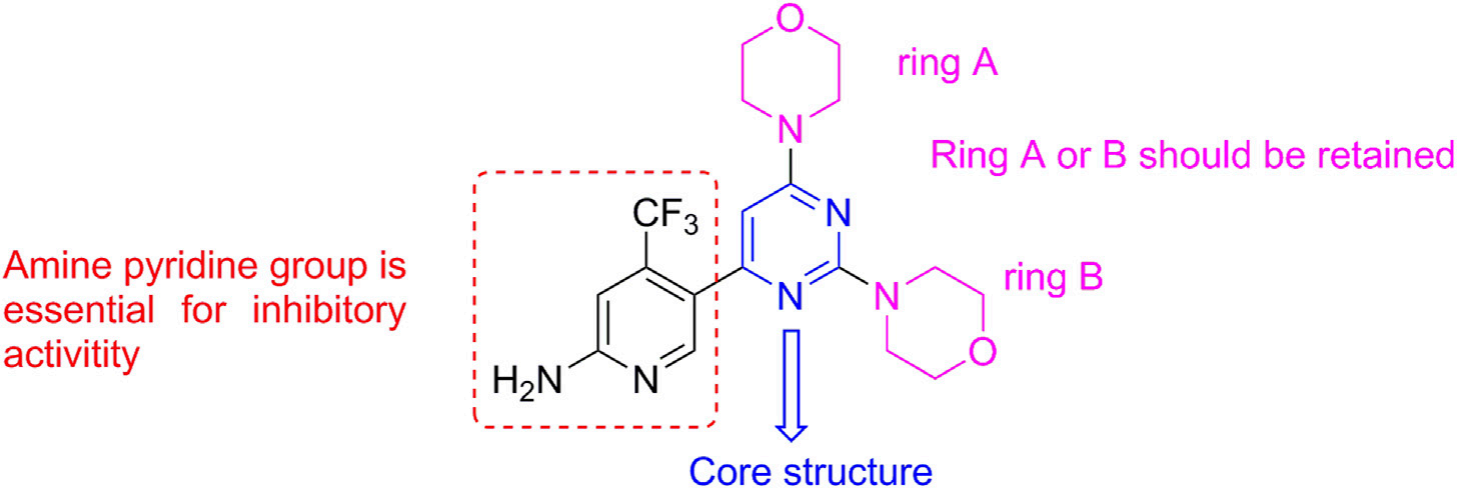
Structural features of the PI3K inhibitor BKM-120.

Most PI3K inhibitors completely bind to the ATP pocket of the protein, which comprises the hinge binder region (key binding site of adenine), the affinity pocket of ribose, and the solvent channel region, related to selectivity (Grommes et al., 2023). Structure–activity relationship (SAR) analysis showed that the morpholine group could form a key hydrogen bond and that the pyrimidine group was the basic framework to maintain the inhibitory activity (Figure 2). The clinical results of the launched drugs were favorable for treating solid tumors; however, drug resistance was also emerging, complicating the interpretation of clinical efficacy (Garces and Stocks, 2018; Wei et al., 2020), which encouraged us to develop a new drug candidate toward the PI3K target.

**FIGURE 3:**
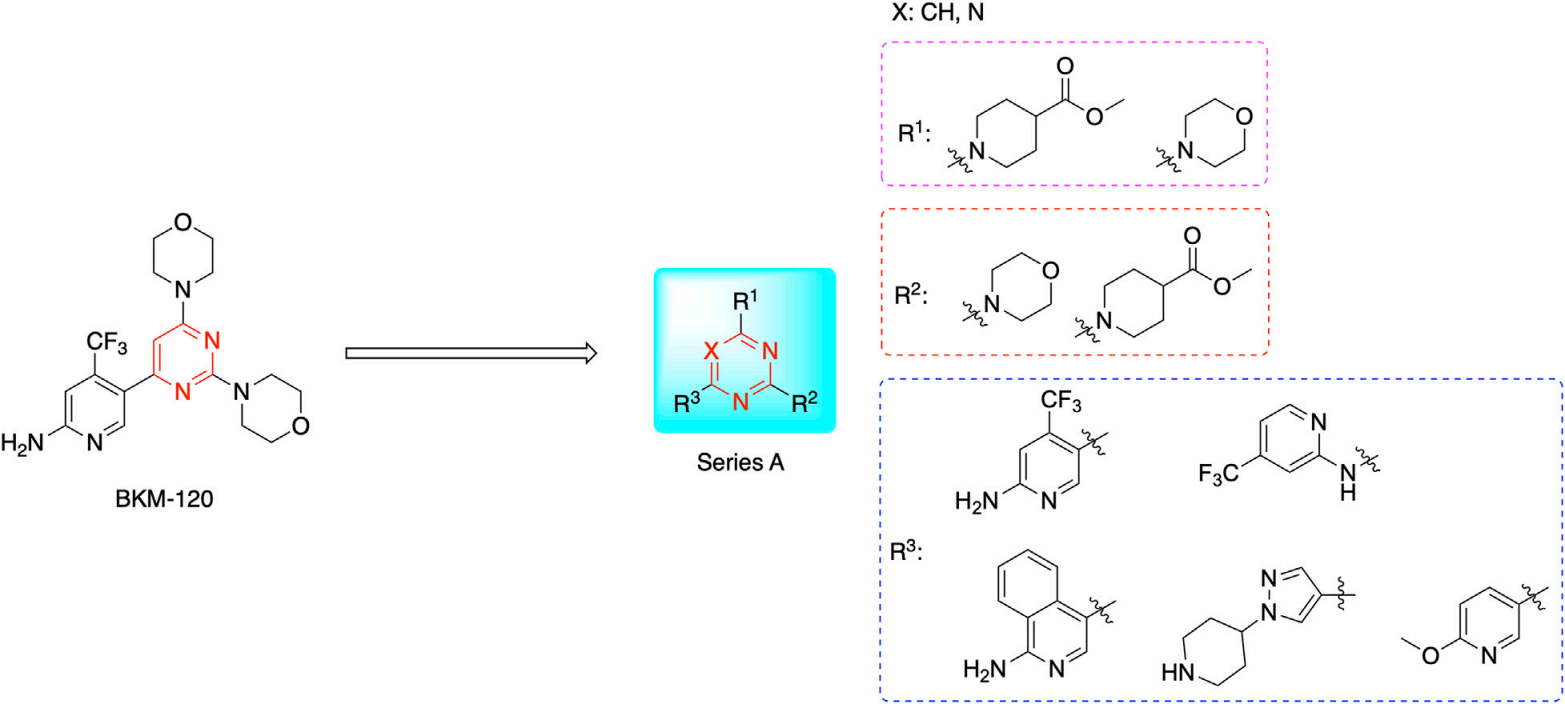
Design strategy and structures of series A compounds.

**FIGURE 4:**
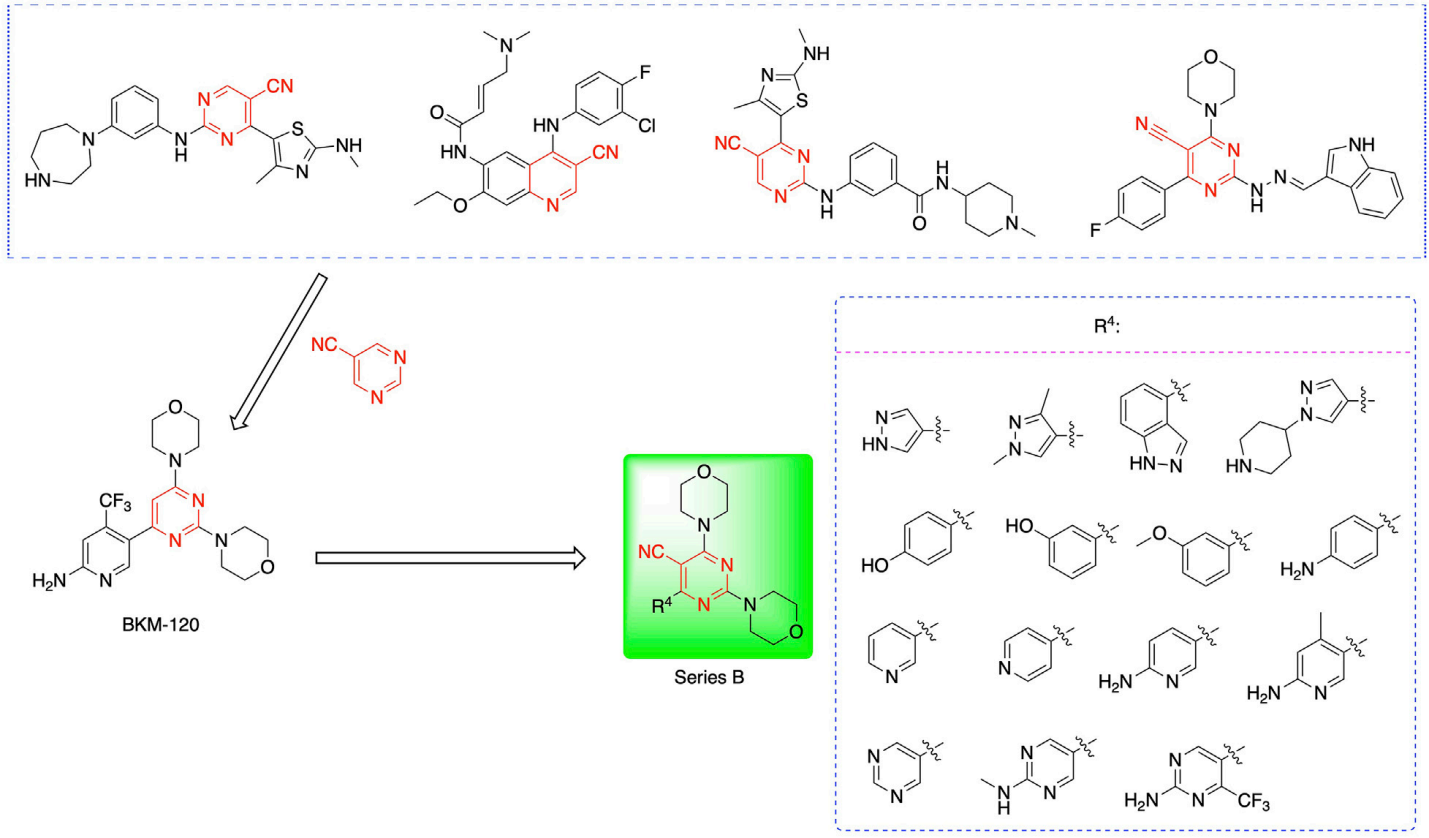
Design strategy and structures of series B compounds.

Most previous SAR studies focused on position 4 or 6 of the pyrimidine scaffolds of BKM-120 (Fairhurst et al., 2022). In this work, we studied series A derivatives formed by substituting position 2, 4, or 6 (Figure 3), but their inhibitory activities were unsatisfactory; thereafter, the pyrimidine-5-carbonitrile fragment, widely used for discovering anti-cancer, anti-inflammatory, and COX-2 candidates (Abdel-Aziz et al., 2021; Nasser et al., 2020; Osman et al., 2022; Helwa et al., 2020), was included as a new core structure instead of a pyrimidine substance (series B; Figure 4) to explore the SAR so as to develop novel PI3K inhibitors with high potency, safety, biological stability, and favorable pharmacokinetic properties.

The target compounds showed similar binding to that of BKM-120, indicating that the pyrimidine-5-carbonitrile scaffold could be a pharmacophore for developing PI3K inhibitors. Compounds 17e, 17m, 17o, and 17p exhibited strong inhibitory activity against PI3Kα, and 17e, 17o, and 17p also showed strong antiproliferative activity against four cancer cell lines (A2780, U87MG, MCF7, and DU145), all comparable to or better than those of BKM-120. In particular, 17p presented strong inhibitory activity against PI3Kδ; significant isoform selectivity against PI3Kβ, PI3Kγ, and mTOR; acceptable safety profiles in normal cells, as well as in the Ames and hERG tests; and favorable pharmacokinetic properties. In an apoptosis assay, 17p also induced significant dose-dependent cytotoxicity in an ovarian cancer cell line (A2780). Thus, we selected it as a candidate for further research and have filed a patent covering these structures (Li et al., 2019).

## 2 Materials and methods

### 2.1 Materials

The reactions were monitored by thin-layer chromatography (TLC) on precoated silica GF_254_ plates. High-resolution mass spectra (HRMS) were taken in the ESI mode using a Waters Q-TOF system. Mass spectra (MSs) were taken in the ESI mode using the Agilent 1100 LC-MS system (Agilent, Palo Alto, CA, United States). ^1^H-NMR and ^13^C-NMR spectra were generated using Bruker AM-400 and 500 spectrometers (Bruker Bioscience, United States) with TMS as the internal standard. The purity of all targeted compounds was confirmed to be >95%, as determined by HPLC using a Waters HPLC instrument with a UV/visible detector by monitoring at 254 nm (column: 5 μm, 4.6 × 250 mm Hypersil ODS-2 column). The mobile phase of the potassium dihydrogen phosphate solution (pH 3.0) and methanol (*v/v*, 3:7) was used with a flow rate of 1.0 mL/min, the ratio was 3:7, and the injection volume was 10 μL. All other chemicals were analytical grade and used without further purification.

### 2.2 Synthetic method

**SCHEME 1:**
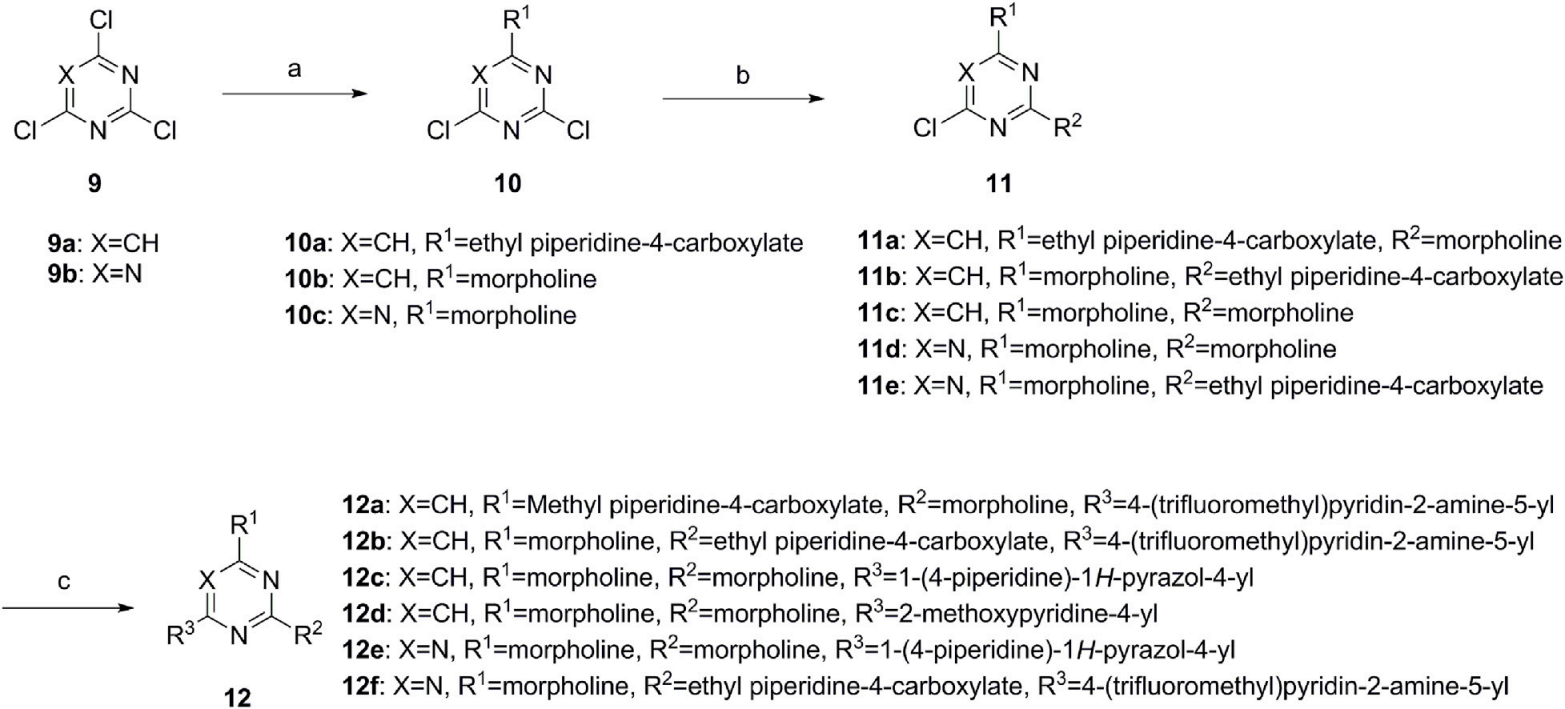
Reagents and conditions: (a) R^1^ group: ethyl piperidine-4-carboxylate or morpholine, TEA, acetone, −20°C; (b) R^2^ group: morpholine or ethyl piperidine-4-carboxylate, DIPEA, NaI, EtOH, 60°C; and (c) R^3^ group: 4-(trifluoromethyl)pyridine-2-amine-5-yl, 1-(4-piperidine)-1*H*-pyrazol-4-yl or 2-methoxypyridine-4-yl, Pd(dppf)_2_Cl_2_, DME, 2N Na_2_CO_3_, 90°C.

**SCHEME 2:**
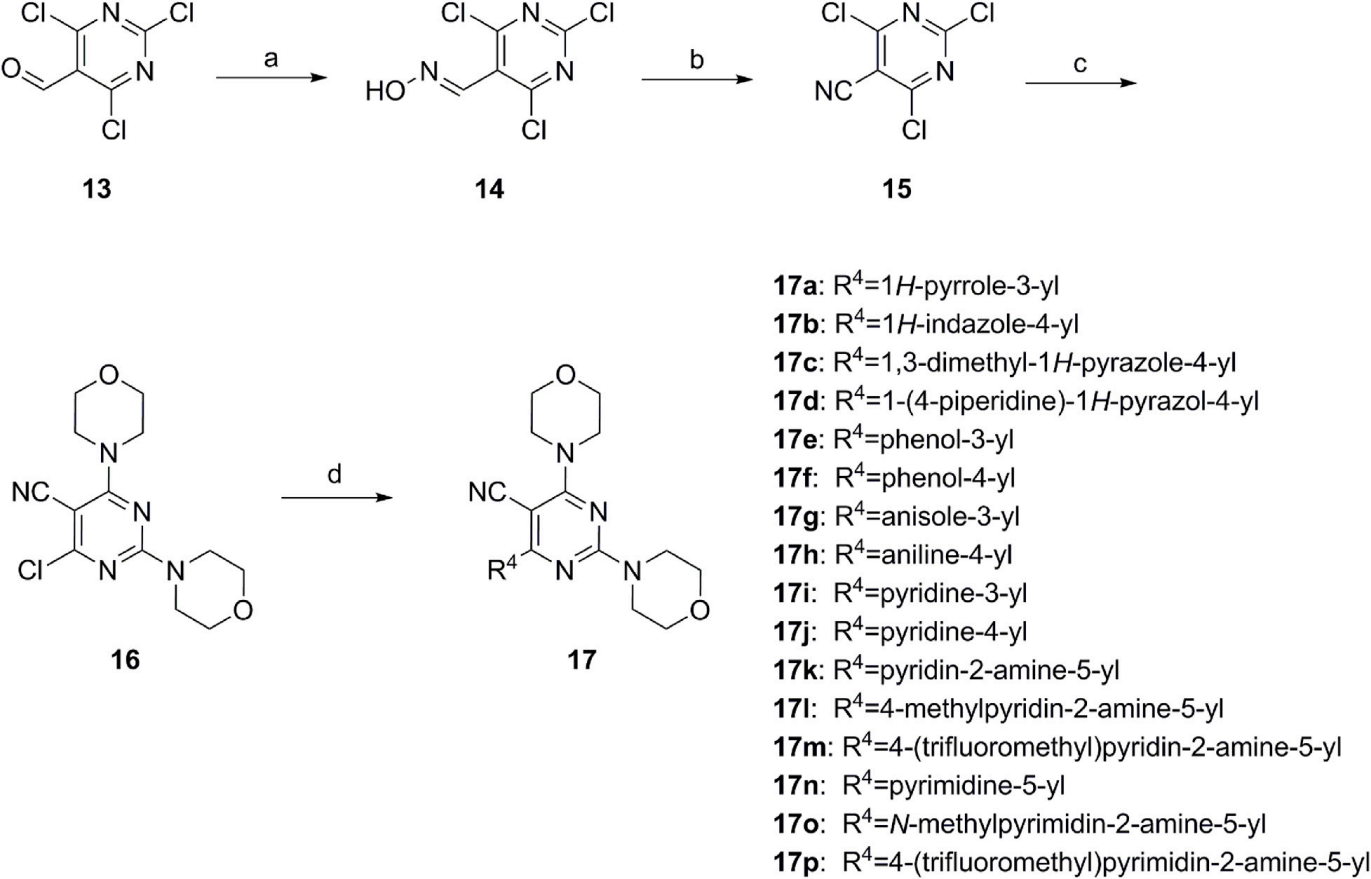
Reagents and conditions: (a) NH_2_OH.HCl, AcOH/H_2_O, r.t.∼60°C; (b) SOCl_2_, reflux; (c) morpholine, THF, −20°C ∼ r.t.; and (d) R^4^ group, Pd(PPh_3_)Cl_2_, K_3_PO_4_, DMF/H_2_O, 110°C.

A series of target compounds and the relative intermediates were synthesized, and the synthetic routes are shown in Scheme 1 and Scheme 2. The structures of the compounds were confirmed by ^1^H-NMR, ^13^C-NMR, and HRMS. Here, 2,4,6-trichloropyrimidine or 2,4,6-trichloro-1,3,5-triazine was allowed to react with the R^1^ group through an S_N_2 reaction to prepare intermediates 10a–10c (Borsari et al., 2019), followed by reacting with the R^2^ group to yield 11a–11e through an S_N_2 reaction (Miller et al., 2013). The products 12a–12f were prepared from 11a to 11e and different boric acid esters through a coupling reaction (Wu et al., 2022). The method is shown in Scheme 1.

First, 2,4,6-trichloropyrimidine-5-carbaldehyde was let to react with hydroxylamine hydrochloride to obtain intermediate 14 (Yang et al., 2023; Pecchi et al., 2010) and was treated with thionyl chloride at reflux to yield the key intermediate 15 (Zhang et al., 2016). The intermediate 16 was obtained by the substitution of chloride with morpholine (Li et al., 2010). Finally, 16 was coupled with substituted boric acid esters to obtain the desired compounds 17a–17p (Dugar et al., 2015). The method is shown in Scheme 2.

### 2.3 Biological method

The *in vitro* inhibition assays of all final compounds against PI3K were performed by Shanghai ChemPartner Co., Ltd. (China). The antiproliferative activities of the compounds against A2780, U87MG, MCF7, and DU145 cell lines were tested by the standard MTT method. CHO cells stably expressing the transcript of hERG were investigated by the automated whole-cell patch clamp technique, using the QPatch system (Sophion). The compound was tested with *Salmonella typhimurium* strains TA98 and TA100 with the plate incorporation method at seven doses. Apoptosis was evaluated using the One-Step TUNEL Apoptosis Assay Kit (FITC) (cat. no. MA0223; Meilunbio, Dalian, China), according to the manufacturer’s protocol. Male and female SD rats (SLRC Laboratory Animal Inc., Shanghai, China) were used, and the detailed pharmacokinetic procedures are described in Supplementary Material.

## 3 Results and discussion

### 3.1 Kinase inhibitory activities and antiproliferative activities

First, the targeted compounds were assayed for their inhibitory activity against PI3Kα, and compounds with good inhibitory activity were further evaluated for their antiproliferative activity against A2780, U87MG, MCF7, and DU145 cancer cells, overexpressing PI3K, using BKM-120 as the positive control.

As shown in Table 1, the inhibitory activities of compounds 12a–12f were poor, and Table 2 shows that some compounds (17e, 17m, 17o, and 17p) had strong inhibitory activity against PI3Kα kinase (IC_50_: 104.1–32.4 nM). In particular, 17o (IC_50_: 34.7 ± 2.1 nM) and 17p (IC_50_: 32.4 ± 4.1 nM) were as potent as BKM-120 (44.6 ± 3.6 nM), and 17e (IC_50_: 88.5 ± 6.1 nM) and 17m (IC_50_: 104.1 ± 12.5 nM) were less potent than BKM-120. These four compounds were further evaluated for their antiproliferative activity against cancer cells. As shown in Table 3, 17e effectively inhibited proliferation in all cell lines as efficiently as BKM-120, while 17o and 17p had a slightly weaker activity than BKM-120, and 17m had weaker antiproliferative activity against A2780 and MCF7 and no inhibitory effects in U87MG and DU145 cells. These *in vitro* results indicated that it is possible to develop novel and effective PI3K inhibitors using the 2,4-dimorpholinopyrimidine-5-carbonitrile group as the pharmacophore.

**TABLE 1:** Inhibitory activities of compounds 12a–12f against PI3Kα.

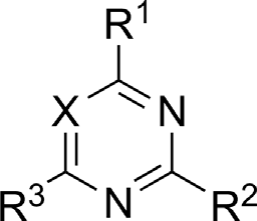					
Compound	X	R^1^	R^2^	R^3^	IC_50_ (nM)^a^
12a	CH	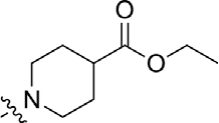	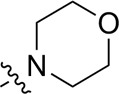	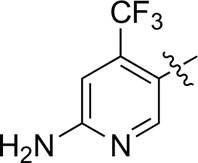	2,287.6 ± 344.3
12b	CH	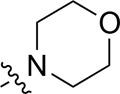	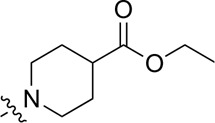	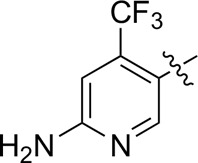	685.4 ± 62.6
12c	CH	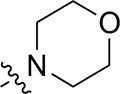	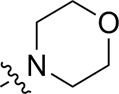	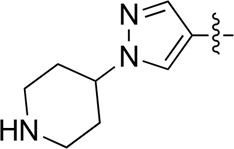	>10,000
12d	CH	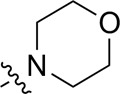	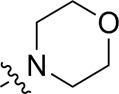	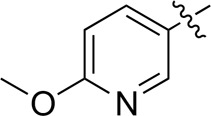	1,007.2 ± 188.9
12e	N	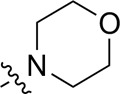	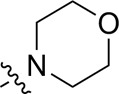	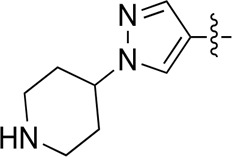	>10,000
12f	N	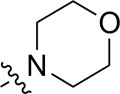	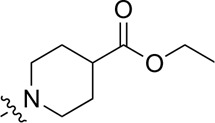	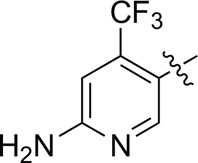	371.3 ± 45.4
BKM-120					44.6 ± 3.6

^a^ Data are means from three independent experiments, in which the variation is less than 20%.

**TABLE 2:** Inhibitory activities of compounds 17a–17p against PI3Kα.

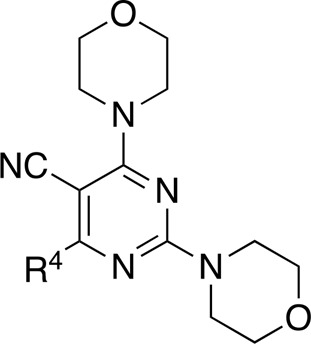					
Compound	R^4^	IC_50_ (nM)^a^	Compound	R^4^	IC_50_ (nM)^①^
17a	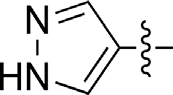	161.8 ± 13.1	17i	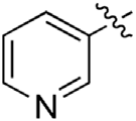	791.8 ± 87.7
17b	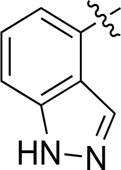	254.2 ± 26.9	17j	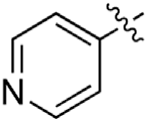	3,036.2 ± 425.8
17c	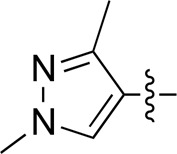	1,357.2 ± 177.3	17k	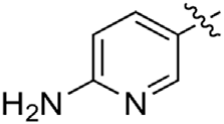	158.7 ± 14.4
17d	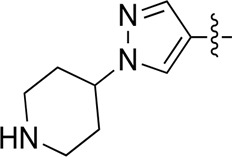	>10,000	17l	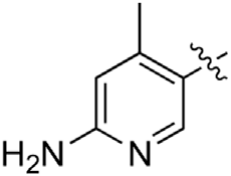	443.2 ± 39.7
17e	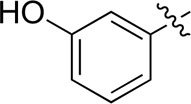	88.5 ± 6.1	17m	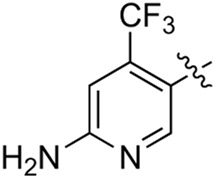	104.1 ± 12.5
17f	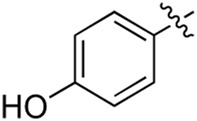	473.2 ± 28.3	17n	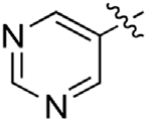	604.3 ± 102.5
17g	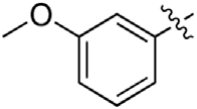	3,272.6 ± 295.2	17o	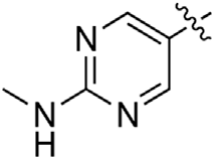	34.7 ± 2.1
17h	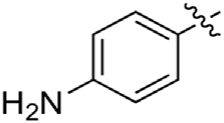	477.2 ± 52.4	17p	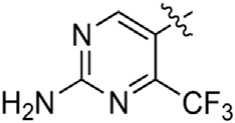	32.4 ± 4.1
			BKM-120		44.6 ± 3.6

^a^ Data are means from three independent experiments, in which the variation is less than 20%.

**TABLE 3:** Cytotoxic activities of compound cell lines *in vitro*.

Compound	IC_50_ (μM)^a^ of four cell lines			
	A2780	U87MG	MCF7	DU145
17e	0.39 ± 0.03	1.75 ± 0.22	0.37 ± 0.03	1.52 ± 0.11
17m	2.72 ± 0.19	66.13 ± 9.94	1.07 ± 0.09	12.36 ± 1.47
17o	1.13 ± 0.10	4.19 ± 0.53	1.16 ± 0.12	5.05 ± 0.47
17p	0.85 ± 0.11	4.72 ± 0.37	1.59 ± 0.08	8.77 ± 1.16
BKM-120	0.41 ± 0.06	2.15 ± 0.19	0.28 ± 0.04	2.24 ± 0.14

^a^ Data are means from three independent experiments, in which the variation is less than 20%.

### 3.2 Preliminary SAR analysis

The pharmacological analysis indicated that the morpholine group played a critical role in PI3K inhibitory activities. The substitution of morpholine decreased PI3Kα inhibitory activity (12a and 12b); thus, the morpholine group was a pharmacophore and should be retained. Next, we designed different R^3^ groups to evaluate inhibitory activities; no activity was observed for 12c, and poor activity was observed for 12d, indicating that the corresponding substituted groups cannot bind with the protein, which may be due to steric hindrance. The triazine compound (12f) had some inhibitory activity (IC_50_: 371.3 ± 45.4 nM), but 12e had no activity. Accordingly, the pyrimidine-5-carbonitrile group was next introduced as a new core structure.

When using a pyrazole or benzopyrazole structure as the R group, the inhibitory activity (IC_50_) of pyrazole was better than that of benzopyrazole (17a > 17b), while the substituted pyrazole compound decreased the inhibitory activity (17c and 17d). When the R group was a substituted phenyl group, the inhibitory activity (IC_50_) of 4-hydroxyl was comparable to that of a 4-amine (17f to 17h) and that of the meta position was better than at a para position (17e > 17f). The substitution of the meta-hydroxyl by a methoxy group decreased the activity (IC_50_/17e > 17g), indicating that the substituted groups affected the binding affinity significantly. In addition, when the R group was a pyridine, the inhibitory activity (IC_50_) of the compound with it at position 3 was better than at position 4 (17i > 17j). When the R group was a substituted pyridine, electron-donating groups decreased the inhibitory activity (IC_50_), while electron-withdrawing groups did the opposite (17m > 17k > 17l). When the R group was a pyrimidine, 2-amine substitution decreased the inhibitory activity (IC_50_), whereas electron-withdrawing groups in position 4 increased it (17p > 17o > 17n).

**FIGURE 5:**
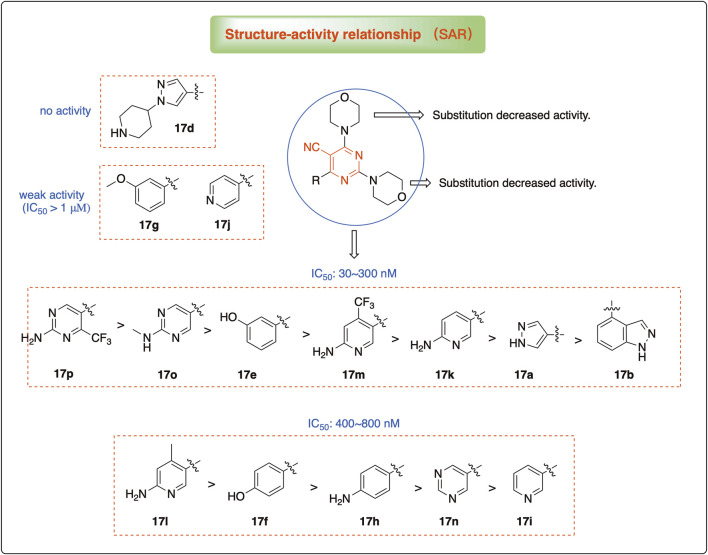
SAR study of the newly designed compounds.

Table 2 shows the activity of compounds 17e, 17m, 17o, and 17p; all were better than others, suggesting their good affinities with PI3K and verifying the design concept. Figure 5 shows the SAR of the newly designed compounds.

### 3.3 Binding model analysis

**FIGURE 6:**
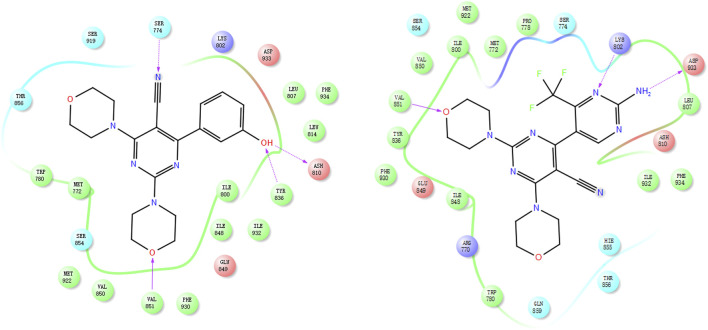
Docking mode of 17e (left) and 17p (right) with PI3Kα (PDB: 3ZIM).

A docking study of compounds 17e and 17p was conducted to assess the rationality of the designed strategy; we selected the co-crystal structure of BMK-120 with PI3Kα (PDB: 3ZIM) as the docking mode. In 17e, the position-2 morpholine group could form a hydrogen bond with Val851, the phenol group could form two hydrogen bonds with Tyr836 and Ash810, and the cyan group could form a hydrogen bond with Ser774 (Figure 6). Similarly, the position-2 morpholine group of 17p could form a hydrogen bond with Val851, and the amine–pyrimidine group could form two hydrogen bonds with Asp933 and Lys802 (Figure 6), which explains its potent activity. These results illustrated that the designed compounds had significant inhibitory potency and that using the 2,4-dimorpholinopyrimidine-5-carbonitrile group as the core structure could effectively inhibit PI3K kinase activity.

### 3.4 Isoform selectivity profile

Compounds 17e, 17o, and 17p were further evaluated for their inhibitory activity against β, γ, and δ isoforms, using BKM-120 as a positive control. Table 4 shows that 17e and 17p could effectively inhibit PI3Kδ (IC_50_: 55.6 ± 3.8 nM and 15.4 ± 1.9 nM, respectively), both better than the positive control (IC_50_: 79.3 ± 11.0 nM). However, compound 17o was weaker (IC_50_: 204.2 ± 22.2 nM). Furthermore, the inhibitory activities of the three compounds against PI3Kβ were comparable and better than that of the positive control. Regarding the inhibitory activity against PI3Kγ, 17e and 17p were worse than the positive control, and 17o was comparable. In summary, 17e and 17p could selectively inhibit PI3Kα and PI3Kδ, thus being selected for further study.

**TABLE 4:** Isoform selectivity study of the selected compounds.

Compound	IC_50_ (μM)^a^				
	PI3Kα	PI3Kβ	PI3Kγ	PI3Kδ	mTOR
17e	86.1 ± 7.8	152.5 ± 18.7	318.2 ± 19.0	55.6 ± 3.8	>10,000
17o	34.7 ± 4.6	176.3 ± 26.3	176.1 ± 15.9	204.2 ± 22.2	203.6 ± 30.1
17p	30.3 ± 3.5	124.7 ± 10.1	286.4 ± 39.6	15.4 ± 1.9	1417.1 ± 184.0
BKM-120	44.6 ± 3.6	279.0 ± 19.2	192.8 ± 23.3	79.3 ± 11.0	>10,000

^a^ Data are means from three independent experiments, in which the variation is less than 20%.

### 3.5 Effects of compounds 17e and 17p on normal cells

Several clinical PI3K candidates were severely intolerable to patients due to fatal hepatotoxicity and, thus, failed in clinical trials. Thus, evaluating the selectivity of compounds toward normal cells might reduce the risk of clinical failure of new compounds. The effects of 17e and 17p were assessed on MRC-5 and HL-7720 cells, using BKM-120 as a control (Table 5).

**TABLE 5:** Inhibitory activity of compounds 17e and 17p on normal cells.

Compound	IC_50_ (μM)^a^			
	MRC-5	HL-7702	A2780	
17e	2.19 ± 0.11	6.17 ± 0.75	0.39 ± 0.03	
17p	8.47 ± 0.77	4.62 ± 0.52	0.85 ± 0.11	
BKM-120	0.98 ± 0.10	0.89 ± 0.06	0.41 ± 0.06	

^a^ Data are means from three independent experiments, in which the variation is less than 20%.

The antiproliferative activities of 17e and 17p on MRC-5 and HL-7702 were weaker than those of BKM-120 and also weaker than the inhibitory activities on A2780, which preliminarily indicated that 17e and 17p had weak toxicity toward normal cell lines MRC-5 and HL-7702, and 17p was selected as a candidate for further research.

### 3.6 hERG test

To evaluate cardiotoxicity, it is necessary to test inhibitory activity on hERG potassium currents. Table 6 shows the IC_50_ value of 17p, and no obvious inhibition of hERG potassium currents was observed, but PI3K activity was inhibited.

**TABLE 6:** Activity of compound 17p on hERG potassium currents.

Compound	IC_50_ (μM)
17p	0.39 ± 0.03
Cisapride	0.41 ± 0.06

### 3.7 The Ames test

*S. typhimurium* TA98 and TA100 were selected to conduct the Ames experiment to investigate the genotoxicity risk of compound 17p. No obvious changes (Table 7) were observed in colony reversion mutation, so compound 17p exerted no mutagenic effect on TA98 and TA100 strains and had no potential genotoxicity risk.

**TABLE 7:** Ames test of compound 17p.

Dosage (μg/dish)	Number of bacterial colony revertant mutations (mean ± SD)	
Bacterial strain	TA98	TA100
Negative	39 ± 6	86 ± 6
3	38 ± 7	88 ± 7
10	41 ± 4	92 ± 3
30	37 ± 11	86 ± 4
100	43 ± 4	91 ± 6
300	29 ± 4	90 ± 3
1,000	44 ± 5	91 ± 1
5,000	40 ± 4	87 ± 1
BKM-120	241 ± 55	555 ± 94

### 3.8 *In vitro* liver microsome stability

The metabolic stability of 17p was evaluated in liver microsome assays, which measure clearance and half-life in rats and humans. The half-life and clearance rates of 17p in human hepatocytes were 127.9 min and 10.8 μL/min/mg, respectively, and 38.5 min and 36.0 μL/min/mg in rat hepatocytes, respectively, indicating that 17p had good *in vitro* liver microsome stability (Table 8).

**TABLE 8:** Microsome stability study of compound 17p.

Compound	RAT			Human		
	T_1/2_ (min)	CL (μL/min/mg)	T_1/2_ (min)		CL (μL/min/mg)	
17p	38.5	36.0	127.9		10.8	

### 3.9 Apoptosis assay

A TUNEL assay showed the number of apoptotic ovarian cancer cells (A2780) under the indicated increasing dose of 17p treatment for 24 h (scale bar: 100 µM). The ED_50_ value of 17p cytotoxicity was determined using a multiple logistic regression model.

**FIGURE 7:**
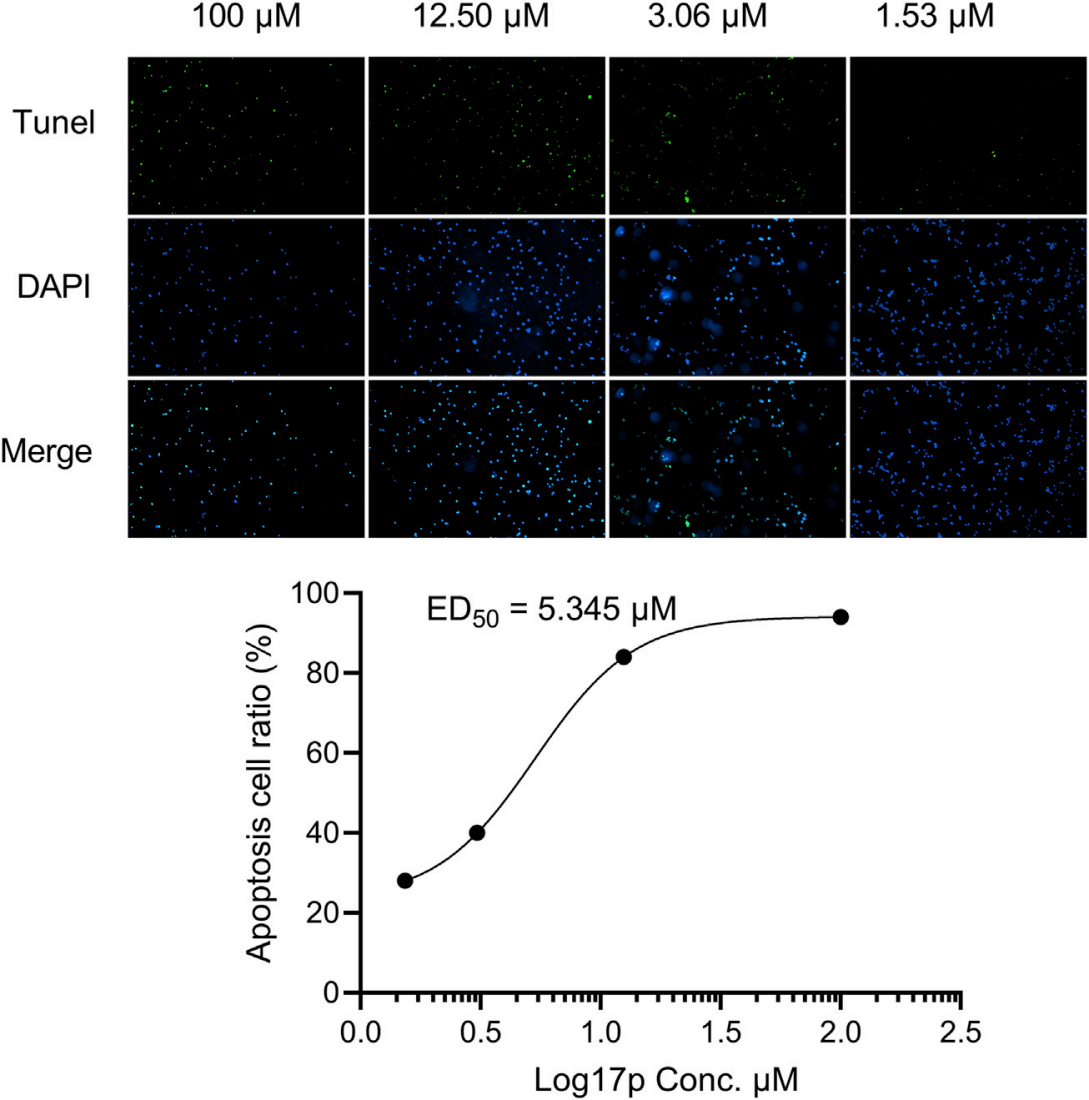
Compound 17p induced cell apoptosis in A2780 cells.

As shown in Figure 7, 17p treatment induced dose-dependent cytotoxicity in A2780 cells. A logistic regression analysis showed a cytotoxicity ED_50_ value of 5.345 µM for compound 17p.

### 3.10 *In vivo* pharmacokinetics

According to the good *in vitro* properties of compound 17p, we performed pharmacokinetic analyses in male and female SD rats at doses of 10 (po) and 2.5 mg/kg (iv), respectively (Table 9). The pharmacokinetic properties in male and female SD rats were extremely variable. In female SD rats with 10 mg/kg through po administration, the area under the curve was 50,618.9 h/ng/mL, the half-life was 2.03 h, and the maximum concentration of 4,860.1 ng/mL was achieved in 3.33 h. The plasma clearance was 0.2 L/h/kg, and the oral bioavailability was 46.2%. Thus, compound 17p had favorable pharmacokinetic properties.

**TABLE 9:** Pharmacokinetic parameters of compound 17p.

M&F	Dose (mg/Kg)	AUC_last_ (h*ng/mL)	T_1/2_ (h)	T_max_ (h)	C_max_ (ng/mL)	Cl__F_pred_ (L/h/kg)	F (%)
M	2.5 (iv)	2,691.4	0.68		3,121.3	0.98	
M	10 (po)	1,857.8	3.19	0.67	537.6	6.54	17.3
F	2.5 (iv)	27,398.2	2.79		4,280.0	0.09	
F	10 (po)	50,618.9	2.03	3.33	4,860.1	0.2	46.2

## 4 Conclusion

In this study, we adopted a pharmacophore-based drug design method to obtain novel 2,4-dimorpholinopyrimidine-5-carbonitrile derivatives that can be used as PI3K inhibitors. Docking studies preliminarily verified the rationality of the structure, owing to the two hydrogen-bonding interactions formed between 17e and 17p with PI3Kα kinase. Furthermore, SAR analyses led to the discovery of a candidate, 17p, with excellent kinase inhibitory activity. In addition, this compound had comparable or better antiproliferative activity against four cancer cell lines than BKM-120. Finally, compound 17p exhibited excellent liver microsome stability and good pharmacokinetic properties, as well as low toxicity in the Ames test and hERG potassium channel assay. Thus, the present findings suggest that compound 17p is worthy of further investigation as a PI3K inhibitor.

## Data Availability

The original contributions presented in the study are included in the article/Supplementary Material; further inquiries can be directed to the corresponding authors.
