# Supplementary material for: Design, synthesis, and biological evaluation of 2,4-dimorpholinopyrimidine-5-carbonitrile derivatives as orally bioavailable PI3K inhibitors
Source: Front Pharmacol. 2024 Oct 21;15:1467028. doi: 10.3389/fphar.2024.1467028 (PMC11533140; doi:10.3389/fphar.2024.1467028)
Supplement: Supplementary file 1 [file DataSheet1.docx]

Supplementary Material

# Experimental

## Chemistry

Reactions were monitored by thin layer chromatography (TLC) on precoated silica GF_254_ Plates. High Resolution Mass spectra (HRMS) were taken in ESI mode on Water Q-Tof. Mass spectra (MS) were taken in ESI mode on Agilent 1100 LC-MS (Agilent, Palo Alto, CA, USA). ^1^H-NMR and ^13^C-NMR spectra were generated on Bruker AM-400 and 500 spectrometers (Bruker Bioscience, USA) with TMS as the internal standard. The purity of all targeted compounds was confirmed to be >95% as determined by HPLC on a Waters HPLC instrument with a UV/visible detector by monitoring at 254 nm (column: 5 μm, 4.6 × 250 mm Hypersil ODS2 column). The mobile phase of potassium dihydrogen phosphate solution (pH 3.0) and methanol (*v/v*, 3:7) was used with a flow rate of 1.0 mL/min, and injection volume was 10 μL. All other chemicals were analytical grade and used without further purification.

## General procedure for Preparation of 2-R^1^-4,6-dichloropyrimidine (triazine) (10a–10c)

To a solution of 2,4,6-dichloropyrimidine (triazine) (0.01 mol) in acetone (30 mL) was added a solution of R^1^ (0.011 mmol) substance and TEA (1.66 g, 0.016 mol) in acetone (20 mL) at **–**20 ^o^C. The reaction solution was stirred at room temperature for 3 h and monitored by TLC. Water (50 mL) was added, and the precipitate was filtered off, washed, and dried in vacuum to yield crude product, which was purified by silica gel chromatography using a mixture of PE/EA (3:1) to afford the product.

1.2.1. Ethyl 1-(2,6-dichloropyrimidin-4-yl)piperidine-4-carboxylate (**10a**)

White solid; 2.59 g, yield: 78.0%, ESI-MS(*m/z*)：304.12[M+H]^+^.

1.2.2. 4-(2,6-dichloropyrimidin-4-yl)morpholine (**10b**)

White solid, 1.79 g, yield: 70.0%, ESI-MS(*m/z*)：234.02[M+H]^+^.

1.2.3. 4-(4,6-dichloro-1,3,5-triazin-2-yl)morpholine (**10c**)

White solid, 1.97 g, yield: 84.0%, ESI-MS(*m/z*)：235.04[M+H]^+^.

## General procedure for Preparation of 2-R^2^-4-R^1^-6-chloropyrimidine (triazine) (11a–11e)

To a solution of the intermediate **10** (4.3 mmol) and R^2^ (4.7 mmol) substance in EtOH (15 mL) was added DIPEA (0.83 g, 6.4 mmol) and NaI (0.064 g, 0.43 mmol). The mixture was stirred at 60 ^o^C for 5 h. Then ethyl acetate (100 mL) was added and washed with water (15 mL×3). The organic phase was dried by anhydrous Na_2_SO_4_ and concentrated to afford intermediate **11**.

1.3.1. Ethyl 1-(6-chloro-2-morpholinopyrimidin-4-yl)piperidine-4-carboxylate (**11a**)

White solid, 1.21 g, yield: 83.0%, ESI-MS(*m/z*): 355.21[M+H]^+^.

1.3.2. Ethyl 1-(4-chloro-6-morpholinopyrimidin-2-yl)piperidine-4-carboxylate (**11b**)

White solid, 0.76 g, yield: 65.0%; MS (ESI) m/z: 355.21[M+H]^+^.

1.3.3. 4,4'-(6-chloropyrimidine-2,4-diyl)dimorpholine (**11c**)

White solid, 0.84 g, yield: 69.0%, MS (ESI) m/z: 285.10 [M+H]^+^.

1.3.4. 4,4'-(6-chloro-1,3,5-triazine-2,4-diyl)dimorpholine (**11d**)

White solid, 0.89 g, yield: 72%, MS (ESI) m/z: 286.10[M+H]^+^.

1.3.5. Ethyl 1-(4-chloro-6-morpholino-1,3,5-triazin-2-yl)piperidine-4-carboxylate (**11e**)

White solid, 1.19 g, yield: 78%, MS (ESI) m/z: 356.27[M+H]^+^.

## General procedure for Preparation of compound 12a–12f

To a mixture of the intermediate **11a**–**11e** (1.0 mmol), boron ester (1.0 mmol) and Pd(dppf)_2_Cl_2_ (10% mol) in DME (10 mL) was Na_2_CO_3_ aqueous (2 N, 3 mL). The mixture was stirred at 90 ^o^C for 10 h under N_2_ atmosphere. The mixture was filtered through celite, followed by concentration to obtain the crude product, which was purified by silica gel chromatography using a mixture of DCM/MeOH (70:1~50:1) to afford the product **12a**–**12f**.

1.4.1. Ethyl 1-(6-(6-amino-4-(trifluoromethyl)pyridin-3-yl)-2-morpholinopyrimidin-4-yl)piperidine-4-carboxylate (**12a**)

White solid, 0.43 g, yield: 63.0%. ^1^H NMR (400 MHz, DMSO-*d*_6_) δ 8.15 (s, 1H), 6.79 (s, 1H), 6.71 (brs, 2H), 6.23 (s, 1H), 4.27 (d, *J* = 13.1 Hz, 2H), 4.07 (q, *J* = 7.1 Hz, 2H), 3.62 (s, 8H), 3.00 (t, *J* = 12.4 Hz, 2H), 2.62 (d, *J* = 11.7 Hz, 1H), 1.87 (d, *J* = 12.9 Hz, 2H), 1.51 (t, *J* = 12.4 Hz, 2H), 1.18 (t, *J* = 7.1 Hz, 3H). ^13^C NMR (101 MHz, DMSO-*d_6_*) δ 179.32, 173.25, 163.44, 160.62, 155.87, 149.72, 143.53, 127.48, 122.54, 114.26, 96.81, 67.55, 54.36, 52.62, 46.97, 38.33, 27.24, 18.57. HRMS: m/z C_22_H_28_F_3_N_6_O_3_ [M+H]^+^ 481.2097, Found 481.2104. Purity: > 97.3% (HPLC). mp 174.6–177.4 ^o^C.

1.4.2. Ethyl 1-(4-(6-amino-4-(trifluoromethyl)pyridin-3-yl)-6-morpholinopyrimidin-2-yl)piperidine-4-carboxylate (**12b**)

White solid, 0.41 g, yield: 60.0%. ^1^H NMR (400 MHz, DMSO-*d_6_*) δ 8.27 (s, 1H), 6.79 (s, 1H), 6.62 (s, 1H), 6.47 (s, 2H), 4.33 (q, *J* = 8.0 Hz, 2H), 3.85 (s, 8H), 3.36**–**3.12 (m, 1H), 2.47 (t, *J* = 7.0 Hz, 1H), 1.89**–**1.77 (m, 4H), 1.34 (t, *J* = 8.0 Hz, 3H). ^13^C NMR (101 MHz, DMSO-*d_6_*) δ 176.35, 169.46, 160.73, 159.76, 157.27, 149.62, 143.28, 126.33, 124.75, 117.71, 98.47, 72.36, 65.32, 57.68, 50.45, 45.52, 28.53, 17.48. HRMS: m/z C_22_H_28_F_3_N_6_O_3_ [M+H]^+^ 481.2097, Found 481.2108. Purity: > 98.3% (HPLC). mp 169.2–173.5 ^o^C.

1.4.3. 4,4'-(6-(1-(piperidin-4-yl)-1*H*-pyrazol-4-yl)pyrimidine-2,4-diyl)dimorpholine (**12c**)

White solid, yield: 71.0%. ^1^H NMR (400 MHz, DMSO-*d*_6_) δ 8.33 (d, *J* = 2.5 Hz, 1H), 8.06 (d, *J* = 2.6 Hz, 1H), 6.46 (d, *J* = 2.6 Hz, 1H), 4.46 (tt, *J* = 10.8, 4.2 Hz, 1H), 3.77**–**3.50 (m, 16H), 3.00 (td, *J* = 12.6, 3.2 Hz, 2H), 2.29**–**1.94 (m, 4H), 1.29**–**1.19 (m, 2H). ^13^C NMR (101 MHz, DMSO-*d_6_*) δ 172.57, 165.32, 163.19, 141.74, 129.43, 116.32, 96.56, 68.77, 61.65, 49.29, 44.24, 28.92. HRMS: m/z C_20_H_30_N_7_O_2_ [M+H]^+^ 400.2383, Found 400.2364. Purity: > 96.8% (HPLC). mp 154.7–157.3 ^o^C.

1.4.4. 4,4'-(6-(6-methoxypyridin-3-yl)pyrimidine-2,4-diyl)dimorpholine (**12d**)

White solid, yield: 62.0%. ^1^H NMR (500 MHz, DMSO-*d_6_*) δ 8.93 (d, *J* = 1.6, 1H), 8.39 (dd, *J* = 8.7, 2.1 Hz, 1H), 6.89 (d, *J* = 8.7 Hz, 1H), 6.69 (s, 1H), 3.91 (s, 3H), 3.76**–**3.59 (m, 17H). ^13^C NMR (126 MHz, DMSO-*d*_6_) δ 165.03, 163.94, 161.60, 160.85, 146.43, 137.91, 127.81, 110.52, 88.71, 66.61, 66.41, 53.87, 44.54, 44.49. HRMS: m/z C_18_H_24_N_5_O_3_ [M+H]^+^ 358.1801, Found 358.1883. Purity: > 98.0% (HPLC). mp 167.8–170.5 ^o^C.

1.4.5. 4,4'-(6-(1-(piperidin-4-yl)-1*H*-pyrazol-4-yl)-1,3,5-triazine-2,4-diyl)dimorpholine (**12e**)

White solid, yield: 56.0%. ^1^H NMR (400 MHz, DMSO-*d*_6_) δ 8.38 (s, 1H), 8.05 (s, 1H), 4.54 (tt, *J* = 9.8, 4.9 Hz, 1H), 3.87**–**3.58 (m, 16H), 3.44**–**3.30 (m, 2H), 3.05 (q, *J* = 11.6 Hz, 2H), 2.27**–**2.11 (m, 4H). ^13^C NMR (101 MHz, DMSO-*d_6_*) δ 178.26, 173.11, 136.85, 129.68, 107.24, 67.56, 63.48, 47.34, 43.26, 28.37. HRMS: m/z C_19_H_29_N_8_O_2_ [M+H]^+^ 401.2335, Found 401.2347. Purity: > 98.3% (HPLC). mp 189.6–192.4 ^o^C.

1.4.6. 5-(4,6-dimorpholino-1,3,5-triazin-2-yl)-4-(trifluoromethyl)pyridin-2-amine (**12f**)

White solid, yield: 35.0%. ^1^H NMR (400 MHz, CDCl_3_) δ 12.09 (s, 1H), 8.70 (s, 1H), 6.82 (s, 1H), 4.98 (s, 2H), 4.69 (s, 2H), 4.15 (q, *J* = 7.1 Hz, 2H), 3.94-3.66 (m, 8H), 3.03 (d, *J* = 11.4 Hz, 2H), 2.64**–**2.50 (m, 1H), 1.97 (dd, *J* = 13.7, 3.9 Hz, 2H), 1.70 (d, *J* = 12.5 Hz, 2H), 1.26 (t, *J* = 7.0 Hz, 3H). ^13^C NMR (101 MHz, DMSO-*d_6_*) δ 178.81, 176.72, 175.25, 171.67, 158.54, 149.25, 143.38, 124.19, 122.83, 112.94, 68.56, 62.27, 54.32, 49.26, 43.83, 27.94, 14.71. HRMS: m/z C_21_H_27_F_3_N_7_O_3_ [M+H]^+^ 482.2049, Found 482.2064. Purity: > 98.4% (HPLC). mp 197.3–200.6 ^o^C.

## Preparation of intermediate (*E*)-2,4,6-trichloropyrimidine-5-carbaldehyde oxime (14)

To a solution of 2,4,6-trichloropyrimidine-5-carbaldehyde (**13**, 5.0 g, 23.65 mmol) in acetic acid (67.8 g, 1.13 mol) and H_2_O (3.30 g, 0.18 mol) was added hydroxylamine hydrochloride (1.64 g, 23.65 mmol). The reaction solution was stirred at 60 ^o^C for 1 h and monitored by TLC. Water (30 mL) was added and the precipitate was filtered off, washed, and dried in vacuum to yield **14** as a yellow solid (4.59 g, 85.7%), which can be used to the next step without any purification.

## Preparation of 2,4,6-trichloropyrimidine-5-carbonitrile (15)

(*E*)-2,4,6-trichloropyrimidine-5-carbaldehyde oxime (**14**, 4.30 g, 18.98 mmol) was added to SOCl_2_ (74.5 g, 0.63 mol) in batch at 0 ^o^C under N_2_ atmosphere. The reaction solution was stirred at reflux for 1 h and monitored by TLC. Then the solution was concentrated to obtain the crude product, which was purified by silica gel chromatography using a mixture of PE/EA (50:1~20:1) to afford the intermediate **15** as a white solid (2.19 g, 55.4%).

## Preparation of 4-chloro-2,6-dimorpholinopyrimidine-5-carbonitrile (16)

To a solution of intermediate **15** (1.50 g, 7.19 mmol) in THF (40 mL) was added the solution of morpholine (2.50 g, 28.76 mmol) in THF (10 mL) at **–**25 ^o^C under N_2_ atmosphere. The mixture was stirred for 1 h at room temperature. The reaction solution was concentrated and poured into water. The precipitate was filtrated and dried to obtain the intermediate **16** as a white solid (1.99 g, 89.64%). ESI-MS(*m/z*): 310.1[M+H]^+^. ^1^H NMR (400 MHz, DMSO-*d*_6_) δ 3.82 (t, *J* = 4.7 Hz, 4H), 3.72 (t, *J* = 4.6 Hz, 4H), 3.68 (dd, *J* = 5.6, 4.0 Hz, 4H), 3.63 (dd, *J* = 5.7, 3.8 Hz, 4H).

## General procedure for Preparation of the title compounds 17a–17p

To a mixture of the intermediate **16** (2.58 mmol), boron ester (3.35 mmol) and K_3_PO_3_ (1.64 g, 7.74 mmol) in DMF (12 mL) and H_2_O (4 mL) was added Pd(PPh_3_)_2_Cl_2_ (0.37 g, 0.52 mmol). The mixture was stirred at 110 ^o^C for 2 h under N_2_ atmosphere. The mixture was filtered through celite, followed by concentration to obtain the crude product, which was purified by silica gel chromatography using a mixture of DCM/MeOH (100:1~40:1) to afford the crude product, followed by stirred with MeOH (10 mL) to get the title compounds **17a**–**17p**.

1.8.1. 2,4-dimorpholino-6-(1*H*-pyrazol-4-yl)pyrimidine-5-carbonitrile (**17a**)

White solid, yield: 42.2%. ^1^H NMR (500 MHz, DMSO-*d_6_*) δ 13.37 (s, 1H), 8.49 (s, 1H), 8.22 (s, 1H), 3.82**–**3.64 (m, 16H). ^13^C NMR (126 MHz, DMSO-*d_6_*) δ 165.13, 163.96, 159.89, 120.48, 119.53, 75.37, 66.34, 47.65, 44.29. HRMS: m/z C_16_H_20_N_7_O_2_ [M+H]^+^ 342.1600, Found 342.1680. Purity: > 99.2% (HPLC). mp 253.7–256.2 ^o^C.

1.8.2. 4-(1*H*-indazol-4-yl)-2,6-dimorpholinopyrimidine-5-carbonitrile (**17b**)

White solid, yield: 12.6%. ^1^H NMR (500 MHz, DMSO-*d_6_*) δ 13.30 (s, 1H), 8.19 (s, 1H), 7.73 (d, *J* = 8.3 Hz, 1H), 7.56 (d, *J* = 6.8 Hz, 1H), 7.50**–**7.45 (m, 1H), 3.89**–**3.65 (m, 16H). ^13^C NMR (126 MHz, DMSO-*d_6_*) δ 171.08, 164.69, 159.84, 140.70, 130.24, 125.87, 122.49, 121.60, 119.50, 113.20, 78.60, 66.39, 47.57, 44.41. HRMS: m/z C_20_H_22_N_7_O_2_ [M+H]^+^ 392.1757, Found 392.1834. Purity: > 99.6% (HPLC). mp 268.3–271.6 ^o^C.

1.8.3. 4-(1,3-dimethyl-1*H*-pyrazol-4-yl)-2,6-dimorpholinopyrimidine-5-carbonitrile (**17c**)

White solid, yield: 70.3%. ^1^H NMR (500 MHz, DMSO-*d_6_*) δ 8.28 (s, 1H), 3.83 (s, 3H), 3.80**–**3.63 (m, 16H), 2.36 (s, 3H). ^13^C NMR (126 MHz, DMSO-*d_6_*) δ 165.26, 165.05, 159.87, 148.49, 133.77, 120.32, 116.71, 76.44, 66.39, 66.35, 47.61, 39.00, 14.76. HRMS: m/z C_18_H_24_N_7_O_2_ [M+H]^+^ 370.1913, Found 370.2000. Purity: > 100% (HPLC). mp 192.5–195.7 ^o^C.

1.8.4. 2,4-dimorpholino-6-(1-(piperidin-4-yl)-1*H*-pyrazol-4-yl)pyrimidine-5-carbonitrile (**17d**)

White solid, yield: 50.0%. ^1^H NMR (600 MHz, DMSO-*d*_6_) δ 8.49 (s, 1H), 8.22 (s, 1H), 4.62 (tt, *J* = 10.3, 5.9 Hz, 1H), 3.98**–**3.51 (m, 16H), 3.40 (dd, *J* = 10.2, 6.5 Hz, 2H), 3.05 (m, 2H), 2.20 (m, 4H). ^13^C NMR (126 MHz, DMSO-*d_6_*) δ 176.64, 168.27, 166.18, 139.19, 130.34, 117.91, 114.26, 80.24, 68.39, 64.81, 50.74, 48.29, 44.62, 27.73. HRMS: m/z C_21_H_29_N_8_O_2_ [M+H]^+^ 425.2335, Found 425.2368. Purity: > 99.4% (HPLC). mp 213.4–215.3 ^o^C.

1.8.5. 4-(3-hydroxyphenyl)-2,6-dimorpholinopyrimidine-5-carbonitrile (**17e**)

White solid, yield: 45.3%. ^1^H NMR (500 MHz, DMSO-*d_6_*) δ 9.69 (s, 1H), 7.33-7.18 (m, 3H), 6.94 (ddd, *J* = 4.1, 3.0, 1.3 Hz, 1H), 3.92**–**3.59 (m, 16H). ^13^C NMR (126 MHz, DMSO-*d_6_*) δ 170.78, 163.72, 158.58, 156.52, 137.78, 128.60, 119.00, 118.48, 117.01, 115.00, 76.75, 65.27, 65.22, 46.51, 43.24. HRMS: m/z C_19_H_22_N_5_O_3_ [M+H]^+^ 367.1644, Found 368.1724. Purity: > 99.7% (HPLC). mp 243.7–246.9 ^o^C.

1.8.6. 4-(4-hydroxyphenyl)-2,6-dimorpholinopyrimidine-5-carbonitrile (**17f**)

White solid, yield: 70.7%. ^1^H NMR (500 MHz, DMSO-*d_6_*) δ 10.08 (s, 1H), 7.75 (d, *J* = 10.3 Hz, 2H), 6.87 (d, *J* = 10.3 Hz, 2H), 4.15**–**3.51 (m, 16H). ^13^C NMR (126 MHz, DMSO-*d_6_*) δ 171.11, 165.29, 160.65, 159.76, 131.39, 128.09, 120.14, 115.42, 76.91, 66.41, 66.37, 47.72, 44.34. HRMS: m/z C_19_H_22_N_5_O_3_ [M+H]^+^ 367.1644, Found 368.1729.Purity: > 95.7% (HPLC). mp 231.2–235.5 ^o^C.

1.8.7. 4-(3-methoxyphenyl)-2,6-dimorpholinopyrimidine-5-carbonitrile (**17g**)

White solid, yield: 59.7%. ^1^H NMR (500 MHz, DMSO-*d_6_*) δ 7.47**–**7.34 (m, 3H), 7.16**–**7.11 (m, 1H), 3.91**–**3.65 (m, 19H). ^13^C NMR (126 MHz, DMSO-*d_6_*) δ 171.65, 164.71, 159.70, 159.41, 138.94, 129.90, 121.69, 119.61, 116.65, 115.01, 78.04, 66.40, 66.36, 55.76, 47.60. HRMS: m/z C_20_H_24_N_5_O_3_ [M+H]^+^ 382.1801, Found 382.1877.Purity: > 96.4% (HPLC). mp 166.2–169.3 ^o^C.

1.8.8. 4-(4-aminophenyl)-2,6-dimorpholinopyrimidine-5-carbonitrile (**17h**)

White solid, yield: 50.0%. ^1^H NMR (500 MHz, DMSO-*d_6_*) δ 7.67(dd, *J* = 8.7, 2.2 Hz, 2H), 6.63 (dd, *J* = 8.8, 2.1 Hz, 2H), 5.76 (s, 2H), 3.84**–**3.56 (m, 16H). ^13^C NMR (126 MHz, DMSO-*d*_6_) δ 170.90, 165.81, 159.80, 152.47, 131.19, 131.19, 123.82, 120.58, 113.11, 66.45, 66.40, 47.83, 44.32, 40.17, 40.17. HRMS: m/z C_19_H_23_N_6_O_2_ [M+H]^+^ 367.1804, Found 367.1878. Purity: > 99.1% (HPLC). mp 202.4–204.7 ^o^C.

1.8.9. 2,4-dimorpholino-6-(pyridin-3-yl)pyrimidine-5-carbonitrile (**17i**)

White solid, yield: 32.7%. ^1^H NMR (500 MHz, DMSO-*d_6_*) δ 8.99 (d, *J* = 1.5 Hz, 1H), 8.74 (d, *J* = 3.7 Hz, 1H), 8.25**–**8.18 (m, 1H), 7.56 (dd, *J* = 7.8, 4.9 Hz, 1H), 3.91**–**3.62 (m, 16H). ^13^C NMR (126 MHz, DMSO-*d_6_*) δ 169.50, 164.12, 159.70, 151.86, 149.86, 136.99, 133.46, 123.83, 119.47, 78.16, 66.37, 47.47, 44.41. HRMS: m/z C_18_H_21_N_6_O_2_ [M+H]^+^ 353.1648, Found 353.1724. Purity: > 99.1% (HPLC). mp 215.4–218.7 ^o^C.

1.8.10. 2,4-dimorpholino-6-(pyridin-4-yl)pyrimidine-5-carbonitrile (**17j**)

White solid, yield: 63.3%. ^1^H NMR (500 MHz, DMSO-*d_6_*) δ 8.76 (d, *J* = 5.8 Hz, 2H), 7.75 (d, *J* = 5.8 Hz, 2H), 3.87**–**3.62 (m, 16H). ^13^C NMR (126 MHz, DMSO-*d_6_*) δ 170.00, 163.98, 159.66, 150.41, 144.89, 123.47, 119.09, 78.14, 66.33, 47.44, 44.40. HRMS: m/z C_18_H_21_N_6_O_2_ [M+H]^+^ 353.1648, Found 353.1711. Purity: > 98.4% (HPLC). mp 241.2–243.8 ^o^C.

1.8.11. 4-(6-aminopyridin-3-yl)-2,6-dimorpholinopyrimidine-5-carbonitrile (**17k**)

White solid, yield: 49.0%. ^1^H NMR (500 MHz, DMSO-*d_6_*) δ 8.52 (d, *J* = 2.0 Hz, 1H), 7.89 (dd, *J* = 8.7, 2.2 Hz, 1H), 6.62 (s, 2H), 6.50 (d, *J* = 8.8 Hz, 1H), 3.89**–**3.61 (m, 16H). ^13^C NMR (126 MHz, DMSO-*d_6_*) δ 169.28, 165.21, 161.88, 159.81, 150.14, 138.11, 121.03, 120.30, 107.35, 76.03, 66.43, 66.39, 47.70, 44.35. HRMS: m/z C_18_H_22_N_7_O_2_ [M+H]^+^ 368.1757, Found 368.1829. Purity: > 97.2% (HPLC). mp 238.1–242.6 ^o^C.

1.8.12. 4-(6-amino-4-methylpyridin-3-yl)-2,6-dimorpholinopyrimidine-5-carbonitrile (**17l**)

White solid, yield: 62.0%. ^1^H NMR (400 MHz, DMSO-*d_6_*) δ 7.96 (s, 1H), 6.33 (s, 1H), 6.28 (s, 2H), 3.81**–**3.72 (m, 8H), 3.72**–**3.60 (m, 8H), 2.20 (s, 3H). ^13^C NMR (101 MHz, DMSO-*d_6_*) δ 177.04, 166.31, 163.28, 159.73, 148.52, 139.85, 126.67, 117.92, 114.93, 78.62, 67.93, 50.71, 48.27, 19.74. HRMS: m/z C_19_H_24_N_7_O_2_ [M+H]^+^ 382.1913, Found 382.2004. Purity: > 97.2% (HPLC). mp 244.3–247.8 ^o^C.

1.8.13. 4-(6-amino-4-(trifluoromethyl)pyridin-3-yl)-2,6-dimorpholinopyrimidine-5-carbonitrile (**17m**)

White solid, yield: 54.5%. ^1^H NMR (400 MHz, DMSO-*d*_6_) δ 8.19 (s, 1H), 6.94 (s, 2H), 6.84 (s, 1H), 3.87-3.53 (m, 16H). ^13^C NMR (101 MHz, DMSO-*d_6_*) δ 178.01, 165.32, 163.28, 158.74, 149.39, 141.82, 124.65, 117.92, 112.94, 77.68, 68.31, 47.28, 45.28. HRMS: m/z C_19_H_21_F_3_N_7_O_2_ [M+H]^+^ 436.1631, Found 436.1712. Purity: > 98.2% (HPLC). mp 247.2–250.4 ^o^C.

1.8.14. 2,6-dimorpholino-[4,5'-bipyrimidine]-5-carbonitrile (**17n**)

White solid, yield: 58.0%. ^1^H NMR (500 MHz, DMSO-*d_6_*) δ 9.36 (s, 1H), 9.24 (s, 2H), 3.95**–**3.63 (m, 16H). ^13^C NMR (126 MHz, DMSO-*d*_6_) δ 166.82, 163.58, 160.12, 159.65, 157.25, 131.58, 119.29, 66.34, 47.36, 44.45, 40.33. HRMS: m/z C_17_H_20_N_7_O_2_ [M+H]^+^ 354.1600, Found 354.1657. Purity: > 96.8% (HPLC). mp 233.7–236.2 ^o^C.

1.8.15. 2'-(methylamino)-2,6-dimorpholino-[4,5'-bipyrimidine]-5-carbonitrile (**17o**)

White solid, yield: 55.0%. ^1^H NMR (400 MHz, CDCl_3_) δ 8.87 (s, 2H), 5.47 (d, *J* = 4.9 Hz, 1H), 4.00**–**3.72 (m, 16H), 3.09 (dd, *J* = 5.0, 3.2 Hz, 3H). ^13^C NMR (101 MHz, DMSO-*d_6_*) δ 177.24, 168.21, 163.17, 161.19, 150.33, 117.92, 113.18, 76.21, 65.37, 46.78, 48.92, 26.25. HRMS: m/z C_18_H_23_N_8_O_2_ [M+H]^+^ 383.1866, Found 383.1944. Purity: > 97.5% (HPLC). mp 239.1–242.8 ^o^C.

1.8.16. 2'-amino-2,6-dimorpholino-4'-(trifluoromethyl)-[4,5'-bipyrimidine]-5-carbonitrile (**17p**)

White solid, yield: 44.3%. ^1^H NMR (500 MHz, DMSO-*d*_6_) δ 8.64 (s, 1H), 7.74 (s, 2H), 3.88**–**3.57 (m, 16H). ^13^C NMR (126 MHz, DMSO-*d_6_*) δ 168.44, 163.68, 162.76, 162.21, 159.32, 152.98, 152.71, 118.95, 117.82, 79.37, 66.36. HRMS: m/z C_18_H_20_F_3_N_8_O_2_ [M+H]^+^ 437.1583, Found 437.1693. Purity: > 96.2% (HPLC). mp 249.3–252.6 ^o^C.

## PI3K inhibitory activity assay

The *in vitro* inhibition assays of all final compounds against PI3K were performed by Shanghai ChemPartner Co., Ltd (China). The inhibitory activity assay of PI3Kα was conducted by Kinase-Glo Luminescent Assay with ATP concentration at 25 μM, while those of PI3Kβ, -γ, and -δ with ADP-Glo Luminescent Assay with ATP concentration at 25 μM. The procedures were as follows: Dilute the compound to 100X of the final desired highest inhibitor concentration in reaction by 100% DMSO. Transfer 100 μL of this compound dilution to a well in a 96-well plate. Transfer 4 μL of compound from source plate to a new 96-well plate as the intermediate plate. Add 96 μL of 1x kinase buffer to each well of the intermediate plate. Mix the compounds in intermediate plate for 10 min on shaker. Transfer 2.5 μL of each well from the 96-well intermediate plate to a 384-well plate in duplicates. Prepare a solution of PI3Kα in 1x kinase buffer at 4-fold the final concentration of each reagent in the assay. Prepare substrate solution of PIP2 substrate and ATP in 1x kinase reaction buffer at 2-fold of the final concentration of each reagent desired in the assay. Cover the assay plate and incubate at room temperature, PI3Kα for 1 h. Equilibrate Kinase-Glo reagent to room temperature. Add 10 μL of Kinase-Glo reagent to each well of the assay plate to stop the reaction Mix briefly with Centrifuge, shake slowly on the shaker for 15 min before reading on a plate reader for luminescence. Finally, collect data on Synergy.

## Antiproliferation Assay

The antiproliferative activities of the compounds against A2780, U87MG, MCF7 and DU145 cell lines were tested by the standard MTT method. Firstly, cells were seeded in 96 well plates, then incubation for 24 h, the cells were exposed to various concentrations of the compounds for addition 72 h. The detailed procedures were described in our previous work. The IC_50_ values were calculated by concentration-response curve fitting using prism 8.

## Binding model analysis

To elucidate the binding mode of the compounds, a detail docking analysis was performed. In our study, the co-crystal structure of BKM-120 with PI3Kα was selected as the docking mode. The inhibitor was docked using the GLIDE docking algorithm in XP (extra-precision) mode.

## hERG potassium currents Assay

CHO cells stably expressing the transcript of hERG were investigated by the automated whole-cell patch clamp technique, using the QPatch system (Sophion). Cells were grown in 175 cm^2^ flasks in serum-supplemented F12 medium in 37 ^o^C 5% CO_2_ incubator. Two days after plating (at 70**–**80% confluence), culture medium was removed and cells were washed with 7 mL PBS (Phosphate Buffered Saline), then cells were treated with 3 mL Detachin for 2 min at 37 ^o^C, followed by the addition of 7 mL serum-free medium, cells were resuspended in the growth medium at a density of 2**–**5*106 cells/mL. The cell suspension was then transferred to the QPatch system, where it was centrifuged; the cells were washed and resuspended in extracellular solution (in mM): 140 NaCl, 5 KCl, 1 CaCl_2_, 1.25 MgCl_2_, 10 HEPES and 10 Glucose, pH 7.4 with NaOH. The composition of the intracellular solution was (in mM): 140 KCl, 1 MgCl_2_, 1 CaCl_2_, 10 EGTA and 10 HEPES, pH 7.2 with KOH. The cells were held at **–**80 mV and activated by a +40 mV pre-pulse of 5 s duration followed by a step to **–**50 mV for 5 s. The voltage protocol is to be repeated every 15 s, the peak of the tail current evoked by the **–**50 mV step was recorded and measured as test parameter. The solution containing the compounds were applied to the cells for 2.5 min following a 5 min baseline recording in extracellular solution. Each cell was received from six escalating concentrations. Each concentration was tested on at least 3 cells. The reference compound cisapride was applied at the end of the test compound addition.

## Ames test

Compound **17p** were tested with *S. typhimurium* strains TA98 and TA100 with the plate incorporation method at seven doses (3, 10, 30, 100, 300, 1000 and 5000 μg/plate). Each dose was tested with and without metabolic activation. The plates were incubated at 37 ^o^C for 72 hours, then calculated as the average of the duplicate plates in the test. The mean number of revertants of compound **17p** and the positive control was compared with that of revertants of the vehicle controls.

## Liver microsome stability Assay

Stock solution (10 mM) of each of test compound was prepared in DMSO. The stock solution for each compound was then diluted into 200 μM with acetonitrile. Incubation mixtures were prepared in a total volume of 200 μL with final component concentrations as follows: 0.1 M PBS (pH 7.4), NADPH (2 mM), liver microsomes (0.2 mg/mL) and test compound (1 μM) or positive control. NAPDH was added after a 5-min preincubation of all other components at 37 ^o^C. Pipette-mixed to achieve a homogenous suspension and immediately transferred 20 μL incubate as a 0 min sample to wells in a “Quenching” plate followed by pipette-mixing. At 5, 15, 30, and 60 min, pipette-mixed the incubate and serially transfer samples of 20 μL incubate per time point to wells in a separate “Quenching” plate followed by pipette-mixing. In “Quenching” plates added 200 μL of acetonitrile with IS. The 96-wells were centrifuged at 4000 rpm for 10 min. 50 μL of supernatant was mixed with 50 μL of ddH_2_O and then injected onto the LC-MS/MS system for analysis.

LC-MS/MS method: Ultra-high performance liquid chromatography-tandem mass spectrometry (UPLC-MS/MS) was applied for quantitation. UPLC was performed using an ACQUITY UPLC Ⅰ-Class Plus system (Waters, Milford, MA, USA). The autosampler was maintained at a temperature of 4 °C for sample injection, and the injection volume was 2 μL. Chromatographic separation was achieved on an ACQUITY UPLC BEH C18 column (2.1 mm × 50 mm, 1.7 µm). The column temperature was maintained at 40 °C. The mobile phase contained 0.1% FA in water for A and 0.1% FA in ACN for B. The flow rate was 0.5 mL/min. The gradient program was as follows: 0–0.2 min, 10% B; 0.2–1.2 min, 10–90% B; 1.2–1.7 min, 90% B; 1.7–1.71 min, 90–10% B; and 1.71–2.2 min, 10% B. Detection and quantification were performed with a Triple Quad 6500 + MS (AB Sciex, Framingham, MA, USA). Processing Software Analyst 1.7.1 was used for data acquisition and processing. Ionization was conducted in positive ion electrospray mode with source temperature of 500 °C, ion spray voltage of 5500 V. MS detection was carried out in MRM mode.

## Apotosis study

Apoptosis was evaluated using One Step TUNEL Apoptosis Assay Kit（FITC） (cat. no. MA0223. Meilunbio, Dalian, China) according to the manufacturer’s protocol. In short, A2780 cells (3 000 per well) were cultured on cover glasses in 24-well plates and treated with **17p** to induce apoptosis. Following 24 h treatment, the cells were fixed with 4% paraformaldehyde and then permeabilized with 0.1% Triton X-100 in PBS. After staining with a TUNEL reaction mixture for 1 h, the cells were counterstained with 1 μg/mL of DAPI for 15 min to visualize cell nuclei, mounted under cover slips with Prolong Antifade Mountant (Beyotime Biotechnology, Wuhan, China). The samples were examined by epifluorescence microscopy, and data are presented as the ratio of TUNEL-positive cells per total amount of cells.

## Pharmacokinetic study

Male and female SD rats (SLRC laboratory Animal Inc., Shanghai, China) were used. For intravenous administration, prepared dosing solution was injected via the femoral vein. The rats were fasted overnight before drug administration and until 6 h after dosing. For the PO experiment, rats (3 in each group) were given a single dose of 10 mg/kg, and heparinized samples of blood were collected at 5, 15, 30 min, 1, 2, 4, 8, and 24 h postdose. For the iv experiment, rats (3 in each group) were given a single 2.5 mg/kg dose, and blood samples were collected at 5, 15, 30 min, 1, 2, and 4 h postdose. Plasma was harvested after centrifugation and stored frozen at 40 oC until analyzed. The concentrations of compounds in plasma were determined by LC/MS/MS (Shimadzu LC-30AD). The results are shown as the maximum plasma concentration (Cmax), the time to reach peak plasma concentration (T_max_), terminal half-life (T_1/2_), and the area under the plasma concentration-time curve from zero to time infinity (AUC_0-inf_).

The method to prepare samples: an aliquot of 10 µL plasma sample was protein precipitated with 100 µL ACN in which contains 20 ng/mL IS. The mixture was vortexed for 10 min and centrifuged at 4000 rpm for 10 min. Transfer 50 µL supernatant to 96 well plates containing 50 µL/vial water. An aliquot of 0.5 µL supernatant was injected for LC-MS/MS analysis.

The HPLC method: Mobile phase A: 0.1%Formic acid in water, Mobile phase B: 0.1%Formic acid in CAN; Wash Solvent: CAN:MeOH:IPA:H_2_O (1:1:1:1)+0.1%FA; Purge Solvent: ACN/Water(v:v=9:1); Column: ACQUITY UHPLC HSS T3 1.8um 2.1*50mm; Oven: 40 ^o^C; Flow rate: 0.600 mL/min.

# HRMS, ^1^H-NMR, ^13^C-NMR and HPLC purity of target compounds

S_1_: HRMS of compound **12d**

S_2_: ^1^H-NMR of compound **12d**

S_3_: ^13^C-NMR of compound **12d**

S_4_: HPLC of compound **12d**

S_5_: HRMS of compound **17a**

S_6_: ^1^H-NMR of compound **17a**

S_7_: ^13^C-NMR of compound **17a**

S_8_: HPLC of compound **17a**

S_9_: HRMS of compound **17b**

S_10_: ^1^H-NMR of compound **17b**

S_11_: ^13^C-NMR of compound **17b**

S_12_: HPLC of compound **17b**

S_13_: HRMS of compound **17c**

S_14_: ^1^H-NMR of compound **17c**

S_15_: ^13^C-NMR of compound **17c**

S_16_: HPLC of compound **17c**

S_17_: HRMS of compound **17e**

S_18_: ^1^H-NMR of compound **17e**

S_19_: ^13^C-NMR of compound **17e**

S_20_: HPLC of compound **17e**

S_21_: HRMS of compound **17f**

S_22_: ^1^H-NMR of compound **17f**

S_23_: ^13^C-NMR of compound **17f**

S_24_: HPLC of compound **17f**

S_25_: HRMS of compound **17g**

S_26_: ^1^H-NMR of compound **17e**

S_27_: ^13^C-NMR of compound **17g**

S_28_: HPLC of compound **17g**

S_29_: HRMS of compound **17h**

S_30_: ^1^H-NMR of compound **17h**

S_31_: ^13^C-NMR of compound **17h**

S_32_: HPLC of compound **17h**

S_33_: HRMS of compound **17i**

S_34_: ^1^H-NMR of compound **17i**

S_35_: ^13^C-NMR of compound **17i**

S_36_: HPLC of compound **17i**

S_37_: HRMS of compound **17j**

S_38_: ^1^H-NMR of compound **17j**

S_39_: ^13^C-NMR of compound **17j**

S_40_: HPLC of compound **17j**

S_41_: HRMS of compound **17k**

S_42_: ^1^H-NMR of compound **17k**

S_43_: ^13^C-NMR of compound **17k**

S_44_: HPLC of compound **17k**

S_45_: HRMS of compound **17p**

S_46_: ^1^H-NMR of compound **17p**

S_47_: ^13^C-NMR of compound **17p**

S_48_: HPLC of compound **17p**
